# Supplementary material for: Conditional chemoconnectomics (cCCTomics) as a strategy for efficient and conditional targeting of chemical transmission
Source: eLife. 2024 Apr 30;12:RP91927. doi: 10.7554/eLife.91927 (PMC11060718; doi:10.7554/eLife.91927)
Supplement: Figure 3—source data 3. [file elife-91927-fig3-data3.zip › Figure 3 source data3 part 1.zip!/Figure 3-Source data 3-part1/1071f2-1-CRISPResso_Report_ztwktvfp.zip!/CRISPResso_on_1071f2_1/9.Alleles_frequency_table_around_sgRNA_gcagaaggatttgtaaatgg.pdf]

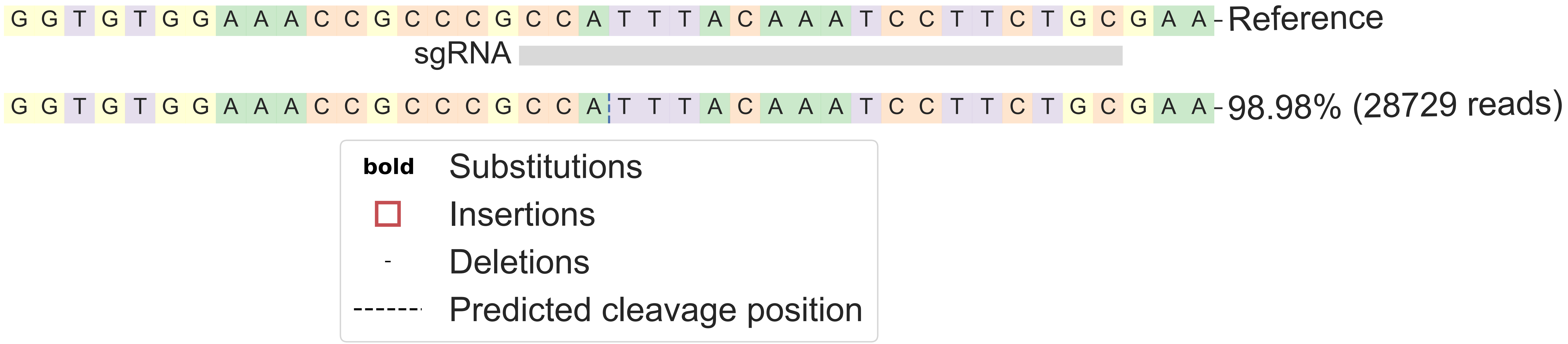
G G T G T G G A A A C C G C C C G C C A T T T A C A A A T C C T T C T G C G A A - Reference
sgRNA
G G T G T G G A A A C C G C C C G C C A T T T A C A A A T C C T T C T G C G A A - 98.98% (28729 reads)
bold Substitutions
Insertions
- Deletions
Predicted cleavage position
